# A Novel Integrating Virtual Reality Approach for the Assessment of the Attachment Behavioral System

**DOI:** 10.3389/fpsyg.2017.00959

**Published:** 2017-06-28

**Authors:** Irene Alice Chicchi Giglioli, Gabriella Pravettoni, Dolores Lucia Sutil Martín, Elena Parra, Mariano A. Raya

**Affiliations:** ^1^Instituto de Investigación e Innovación en Bioingeniería (i3B), Universitat Politécnica de ValenciaValencia, Spain; ^2^Department of Oncology and Hemato-Oncology (DIPO), University of MilanMilan, Italy; ^3^Applied Research Division for Cognitive and Psychological Science, European Institute of OncologyMilan, Italy; ^4^Departamento de Economía de la Empresa, Facultad de Ciencias Sociales y Jurídicas, Universidad Rey Juan CarlosMadrid, Spain

**Keywords:** attachment, virtual reality, presence, ecological validity, stealth assessment, evidence-centered design

## Abstract

Virtual reality (VR) technology represents a novel and powerful tool for behavioral research in psychological assessment. VR provides simulated experiences able to create the sensation of undergoing real situations. Users become active participants in the virtual environment seeing, hearing, feeling, and actuating as if they were in the real world. Currently, the most psychological VR applications concern the treatment of various mental disorders but not the assessment, that it is mainly based on paper and pencil tests. The observation of behaviors is costly, labor-intensive, and it is hard to create social situations in laboratory settings, even if the observation of actual behaviors could be particularly informative. In this framework, social stressful experiences can activate various behaviors of attachment for a significant person that can help to control and soothe them to promote individual’s well-being. Social support seeking, physical proximity, and positive and negative behaviors represent the main attachment behaviors that people can carry out during experiences of distress. We proposed VR as a novel integrating approach to measure real attachment behaviors. The first studies on attachment behavioral system by VR showed the potentiality of this approach. To improve the assessment during the VR experience, we proposed virtual stealth assessment (VSA) as a new method. VSA could represent a valid and novel technique to measure various psychological attributes in real-time during the virtual experience. The possible use of this method in psychology could be to generate a more complete, exhaustive, and accurate individual’s psychological evaluation.

## Introduction

During experiences of distress, people of all ages can carry out various behaviors, such as social support seeking, physical proximity, and positive and negative behaviors. Experiences of loneliness, separation, threat, and loss can activate interpersonal behaviors of attachment for a significant person that can help to control and soothe them to promote individual’s well-being (Schmidt et al., 2002, 2003; Maunder et al., 2005, 2006; Ditzen et al., 2008).

Attachment paradigm represents one of the models that attempted to explain the formation of social and close relationship patterns. According to Bowlby (1969) the quality of the first parent–child interactions shape the cognitive and emotional representations that will later drive the self and other’s perceptions, and new relationships (Bowlby, 1969, 2005; Main et al., 1985; Ainsworth et al., 2015). In attachment research, the assessment methods widely used and validated are self-report questionnaires, interviews or projective measures (see the review of Ravitz et al., 2010), while attachment behaviors have been addressed less in adults. The observation of attachment behaviors is costly, labor-intensive, and it is hard to create social situations in laboratory settings, however, observation of actual attachment behaviors could be particularly informative.

In the current article, we suggest virtual reality (VR) as a novel integrating approach to elicit attachment behavioral system, as well as a powerful tool to bridge the gap among realism, dynamics of social interactions and experimental control. Indeed, VR can generate real-life simulated situations, including many sensory simulators, such as stereo headphones, olfactory and haptic devices, which can create real immersive world experiences. Furthermore, VR can provide several advantages in the assessment of attachment behavioral system. First, VR can create various stimuli, which can target specific aspects of attachment system (e.g., support seeking, physical proximity, positive and negative behaviors). Second, VR can be used to track behavioral and physiological responses, allowing an integrated assessment of the attachment experiences. Within the VR approach, we suggest stealth assessment as new method for assessing the attachment behavioral system since it can incorporate assessment of multi-attachment behaviors in real time during the virtual experience.

## Virtual Reality as a Novel Integrating Tool for Assessing

Virtual reality can be defined as a simulated experience formed by a synthetic computer-based environment, able to create the sensation of undergoing real experiences. Users become active participants and interact with the virtual situations and other virtual agents, seeing, hearing, and feeling the experience as if they were acting in the real world (Wilson, 2002; Shapiro, 2007).

Until now, VR has showed its efficacy in clinical psychology, neuropsychology, and in cognitive and motor rehabilitation (Llorens et al., 2014, 2016; Borrego et al., 2016). In clinical psychology, VR has been especially applied for the treatment of different anxiety disorders (e.g., phobias) (Botella et al., 2006; Wrzesien et al., 2014), mood disorders (Baños et al., 2006; Meyerbröker and Emmelkamp, 2010), post-traumatic stress disorder (PTSD) (Rothbaum et al., 2001; Cárdenas-López and Rosa-Gómez, 2011; Cárdenas and De La Rosa, 2012), and body image disturbances in patients with eating disorders (Riva et al., 2001, 2016; Gorini et al., 2010; Ferrer-García and Gutiérrez-Maldonado, 2012; Dakanalis et al., 2016). In VR clinical treatments, patients are exposed to fear situations or specific traumatic events to gradually face in a safe and controlled environment. Recently, VR has become an important tool for simulating real world events, allowing moving the subject to a virtual world where any kind of stimulus is possible. Furthermore, VR treatments rely on previous assessments that inform diagnosis. Usually, the assessment uses paper and pencil tests, providing people’s conscious answers and attitudes about themselves and others at a given time.

Even if this assessment approach provides highly systematic validity and reliability, they present some limitations in terms of detecting the unware responses, as well as in creating and experiencing real situations able to activate specific behaviors related to the construct that we want to assess. Furthermore, they have also been criticized as limited in the ecological validity, referring to the degree of correspondence that a test has relative to the real world or real behaviors in context or situation.

In this framework, VR could advocate as a powerful assessing tool to improve ecological validity, maintaining experimental control, and to approach research themes that could be answered together with the traditional method, thanks to the technical capacity needed to approach behavioral topics.

In the next paragraph, we deepen the main features of VR to elicit a stronger and more real experience and to obtain reliable measurements of human behavior.

### Virtual Reality Features

Immersion, interactivity, realism, and transference represent the most features of VR experiences (Biocca, 1997; Lombard and Ditton, 1997; Loomis et al., 1999; Heeter, 2000; Biocca et al., 2001; Bailenson et al., 2006; Saribay and Andersen, 2007; Skalski and Tamborini, 2007; Andersen and Thorpe, 2009; Slater, 2009; Sundar et al., 2010; Cummings and Bailenson, 2016). Immersion can be described as a technological feature based on various input and output devices able to produce sensory and perceptual information that create the illusion of being in the physical world (Slater, 2009). The combination of more sensory and perceptual information with stereoscopic visual input (e.g., Head Mounted Display – HMD), audio and tactile input, can augment the level of immersion, generating depth and perception of realism (Loomis et al., 1999; Cummings and Bailenson, 2016). In literature, higher level of immersion, compared to lower (e.g., desktop computer) reported significant results in terms of explorative tasks (Boyd, 1997; Sandstrom et al., 1998), distance estimation and depth perception (Heineken and Schulte, 2001), cooperative tasks (Roberts et al., 2005), and short-term spatial memory (Adamo-Villani and Johnson, 2010; Bailey et al., 2012). Interactivity can be defined as the perception process in which user can interact with virtual contents and agents, in which the virtual contents and agents respond to the user’s actions in real-time, providing high level of engagement and realism (Lombard and Ditton, 1997; Heeter, 2000; Biocca et al., 2001; Skalski and Tamborini, 2007; Sundar et al., 2010). Furthermore, as mentioned VR allows social interactions among two or more virtual or real agents through verbal or written information, such as voice or video recordings, human actors, and vignettes, and non-verbal behaviors, such as mutual gaze or interpersonal distance. Studies on the effectiveness of social interactions in VR showed that people transfer their emotions behaviors, and expectations of past relationship to new relationships, specifically to virtual agents (Sanchez-Vives and Slater, 2005; Bailenson et al., 2006; Yee and Bailenson, 2007; Schönbrodt and Asendorpf, 2010, 2011). More in detail, Schönbrodt and Asendorpf (2010) examined the correlation between the behaviors for a virtual partner and real-life relationships measuring variables as interpersonal motives and relationship satisfaction. Results showed that participants transferred their emotional and behavioral patterns to the virtual partner as if he/she was the real partner. At the same time, those were dissatisfying with their real relationship, initially, projected their negative expectations on the virtual character, and during the game tried to re-establish close and positive interactions.

These features can create the sensation of presence, the subjective psychological state of “being there” in the virtual world leaving the perception of technological mediation behind (Biocca, 1997; Lombard and Ditton, 1997; Persky and Blascovich, 2008).

Finally, VR can create any kind of stimulus, also reproducing the same stimuli infinite times, allowing an extensive control over the measurement validity.

### Behavioral and Physiological Measures in Virtual Reality

Virtual reality can also integrate behavioral and physiological measures for tracking users’ responses. Indeed, VR allows measuring users’ behavior during the experience, using motion-tracking device systems, such as eye tracking devices, or head-movement devices. These devices can track head-movements and eye-movements, allowing the analysis of non-verbal displays of attachment. In addition, inside a virtual environment, physiological measures can be monitored by wearable device. Heart rate variability, skin conductance, and skin temperature can also be tracked during the virtual experience, providing continuous and real-time data. Therefore, VR can be seen as an integrated measurement tool providing more ecological and controlled setting able to develop a more holistic and comprehensive psychological model of the experiences (Loomis et al., 1999; Tarr and Warren, 2002; Bohil et al., 2011; Parsons, 2015; Fusaro et al., 2016).

## Applying Virtual Reality for Assessing Attachment Behavioral System

### Attachment Theory

Attachment theory is a composite psychosocial development theory that explains the formation of interpersonal relationships patterns. Bowlby (1969) theorized that children have a natural predisposition to develop a bond of attachment to a significant other, the attachment figure, especially in stressful situations. During these experiences, children aim to seek and maintain proximity to the attachment figure, looking for support and safety (Bowlby, 1969). If the attachment figure gives attentive and positive emotional responsiveness, the child will build positive mental representations of self and others (Gunnar et al., 1996) and vice-versa. The positive or negative mental representations formed internal working models (IWMs) about self, others, and relationships, allowing later to regulate behaviors based on formed expectations. In this framework, several longitudinal studies have shown that the IWMs remain quite stable during the lifespan (Stern, 1985; Tronick and Gianino, 1986; Beebe and Lachmann, 1994; Beebe et al., 1997; Hamilton, 2000; Waters et al., 2000; Steele et al., 2003; Weinfield et al., 2004; Sroufe, 2005). In this framework, a longitudinal study of Sroufe et al. (2009) has showed a continuity and stability over the time among the attachment behaviors. More in detail, children with positive mental representations will be adults able to react to stressful events with lower levels of distress and are more likely to cope with stress by seeking support, hold positive expectations about relationships. Instead, children with negative mental representations will be adults that can tend, on one hand, to perceive others as unresponsive or inconsistent, worry about being rejected, showing hyper-activation strategies and hypervigilance (Main, 1990; Collins et al., 2006; Ainsworth et al., 2015). Hyper-activation strategies demand proximity and care from attachment figures (Mikulincer et al., 2003) including hypervigilance toward attachment cues, use of worry, and rumination (Cassidy, 1994). These strategies often fail and can amplify distress (Mikulincer and Shaver, 2007). On other hand, individuals with negative mental representations can prefer being distant and detached from others, report no need for close relationships, and tend to distrust affective signals from others (Cassidy and Kobak, 1988; Mikulincer et al., 2003).

### Research on Virtual Reality for Assessing Attachment Behavioral System

To date, three studies have investigated the attachment behavioral system in close relationships using virtual environments (Kane et al., 2012; Schönbrodt and Asendorpf, 2012; Symons et al., 2015). In the first study, Schönbrodt and Asendorpf (2012) created a 2D online computer game (Simoland), for investigating attachment behaviors toward a virtual partner during attachment-related experiences (separation, conflict, and threat). Technologically, Simoland used a low-cost and open environment with non-immersive graphical representation in which the characters were depicted by simple beings able to interact each other by vignettes. The participant could direct the behaviors (physical distance, support seeking, positive and negative behaviors) of one virtual character. Furthermore, during the game, the participant attributed the emotions of the virtual character. Results showed significant correlation between the participant attachment style and virtual character behaviors across the three conditions. More in detail, across the three conditions, anxious participants showed more negative emotional behaviors and responses, looking for more physical proximity and support seeking than avoidant participants that, they acted by using more deactivating strategies.

In the second study, Kane et al. (2012), using a 3D-immersive VR environment, hypothesized that the presence of a partner was not enough for providing individual’s safety. They created a canyon path in which participants had to walk, seeing or not seeing at the end of the path their virtual partner. The authors compared three conditions: in the first condition participants should walk alone for the canyon; in the second condition the virtual partner at the end of the path was bodily oriented to the participant; in the third condition, the virtual partner was not oriented to the participant. Results showed that participants in the second condition perceived the walking task as less stressful than those who were alone and more secure than those in the third condition. Furthermore, participants in the third condition showed later less physical proximity from their partner.

The third and more recent study (Symons et al., 2015) examined relationship between adult attachment styles and caregiver attitudes toward a virtual personalized child, raised in a simulated parenting environment from birth to 19 years. The results showed that avoidance and anxiety attachment styles were negatively related to caregiver attitudes. More in detail, dismissing and preoccupied adults showed less positive attitudes toward a child and felt less willingness as an attachment figure to their child.

These three studies, even if considered different aspects of the attachment system, they used a novel methodology: the simulated VR experience, showing significant results consistent with the previous studies that used traditional methods of assessing (Ainsworth, 1978; Mikulincer and Shaver, 2005). However, these studies showed how the attachment paradigm is a broad and complex construct, consisting of multi-aspects. Indeed, if Schönbrodt and Asendorpf (2011) have correlated behavioral measures, such as interactional actions (positive vs. negative), emotional attribution, and physical distance with attachment and emotional scales, Kane et al. (2012) have correlated *ad hoc* measures about stress appraisal, emotional security, and perceived responsiveness of self, while Symons et al. (2015) utilized measures about caregiver attitudes, secure scale, and trait anxiety questionnaires. Interesting and novel behavioral measures have been introduced by Kane et al. (2012). More in detail, vigilance and interpersonal distancing behaviors have been measured to obtain more accurate implicit measures of social approach/avoidance. As noted above, the possibility to introduce behavioral measures for the assessment of the attachment system can represent a potential of VR together with the traditional methods.

### Virtual Stealth Assessment Method for Assessing Attachment Behavioral System

Virtual stealth assessment (VSA) can be defined as a new real-time performance-based assessment that allows assessing individuals’ attributes (Shute, 2009). SA, introduced in virtual learning games, allows directly and invisibly assessing various behaviors related to attributes while users play, correlating them to traditional assessment (pre-and posttests) (Shute and Ke, 2012). The development of a VSA is based on evidence-centered design (ECD), characterized by three models of reference: the competence model is based on the recognition of which attributes we want to assess, the structural model in which the behaviors that could reveal the attributes are identified, and the task model in which are developed tasks that could elicit those behaviors previously described Mislevy et al. (2003). An example of stealth assessment can be represented by Newton’s Playground, a 2D computer-game, constituted by 74 problems of increasing difficulty, which aim for the user was to lead a green ball to a red ball for blowing up it. To move the ball, the users had to draw or create inclined ramps, pendulums, levers, and springboards, according to the rules of Newton’s gravity. The game assessed three competencies: creativity, conscientiousness, and conceptual physics understanding (Shute et al., 2013). For example, according to the literature, consciousness is formed by several facets, such as persistence, perfectionism, organization, and carefulness. For each component various behaviors are identified with the relative development of the tasks or problems for solving. As in Newton’s Playground, we propose the implementation of VSA for the evaluation of the attachment behavioral system. The focus will be on assessing behavioral aspects related to attachment, such as physical distance, support seeking, positive and negative behaviors in stressful and non-stressful situations. The development of VSA will be based on creating an immersive social videogame, involving five conditions, that they will include stressful social simulated experiences of loneliness, separation, threat, and loss, more non-stressful social situations. For each situation, several social interaction decisions making and problem solving tasks will be implement in the virtual environment in order to assess the behaviors mentioned above. In accordance with ECD, the assessment will be woven into the environment and invisible to user, encouraging the elicitation of real behaviors, for later correlating outcome behaviors with the traditional attachment questionnaires. VSA would provide a new method of assessment that, integrated with the traditional paper and pencil tests, could enrich assessment.

## Conclusion

The attachment system is a composite paradigm for understanding relationship processes, traditionally assessed by valid and reliable interview, projective, or self-report measures (Ravitz et al., 2010), but with a limited ecological validity in the assessment of real attachment behaviors. VR and SA provide a new approach and method to measure them in real-time, allowing to simulate various attachment situations, maintaining high levels of experimental control and ecological validity. Several studies support VR as a treatment technology (Baños et al., 2006; Meyerbröker and Emmelkamp, 2010; Cárdenas-López and Rosa-Gómez, 2011; Wrzesien et al., 2014; Riva et al., 2016) but not for the assessment, mainly based on paper and pencil tests. SA will allow for a multi-level assessment of attachment that could be useful to catch real behaviors during the virtual experience (Shute, 2009). Finally, the possible use of this method in psychology could be to generate a more complete, exhaustive, and accurate individual’s psychological evaluation.

## Author Contributions

All authors have significantly contributed to the manuscript according to the specific scientific competences. MR and IC made substantial contributions to manuscript conception. IC participated in drafting the manuscript, while DS, GP, and EP revised it critically for important intellectual content. MR and GP supervised the entire work. All authors give final approval of the version to be submitted and any revised version.

## Conflict of Interest Statement

The authors declare that the research was conducted in the absence of any commercial or financial relationships that could be construed as a potential conflict of interest.
